# Assumptions on decision making and environment can yield multiple steady states in microbial community models

**DOI:** 10.1186/s12859-023-05325-w

**Published:** 2023-06-22

**Authors:** Axel Theorell, Jörg Stelling

**Affiliations:** Department of Biosystems Science and Engineering (D-BSSE) and SIB Swiss Institute of Bioinformatics, ETH Zurich, 4058 Basel, Switzerland

**Keywords:** Microbial communities, Flux balance analysis, Game theory

## Abstract

**Background:**

Microbial community simulations using genome scale metabolic networks (GSMs) are relevant for many application areas, such as the analysis of the human microbiome. Such simulations rely on assumptions about the culturing environment, affecting if the culture may reach a metabolically stationary state with constant microbial concentrations. They also require assumptions on decision making by the microbes: metabolic strategies can be in the interest of individual community members or of the whole community. However, the impact of such common assumptions on community simulation results has not been investigated systematically.

**Results:**

Here, we investigate four combinations of assumptions, elucidate how they are applied in literature, provide novel mathematical formulations for their simulation, and show how the resulting predictions differ qualitatively. Our results stress that different assumption combinations give qualitatively different predictions on microbial coexistence by differential substrate utilization. This fundamental mechanism is critically under explored in the steady state GSM literature with its strong focus on coexistence states due to crossfeeding (division of labor). Furthermore, investigating a realistic synthetic community, where the two involved strains exhibit no growth in isolation, but grow as a community, we predict multiple modes of cooperation, even without an explicit cooperation mechanism.

**Conclusions:**

Steady state GSM modelling of microbial communities relies both on assumed decision making principles and environmental assumptions. In principle, dynamic flux balance analysis addresses both. In practice, our methods that address the steady state directly may be preferable, especially if the community is expected to display multiple steady states.

**Supplementary Information:**

The online version contains supplementary material available at 10.1186/s12859-023-05325-w.

## Background

Microbial communities perform essential functions in diverse environments such as the soil [1] and the human gut [2]. While the experimental characterization of community composition is relatively easy with metagenomics methods, this is not true for the analysis of functional metabolic interactions between community members [3]. The paradigm of constraint based modelling of metabolism with genome scale models (GSMs) [4] has therefore become increasingly popular for the analysis of microbial communities [5, 6]. For example, a recent GSM-based study stipulated that whether a microbial community is cooperative or competitive correlates strongly with the nutrient abundance in its natural habitat [7].

Approaching community functions with GSMs requires two key ingredients: models and simulation methods. Models are no longer a main limitation because of the ease with which large, organism-specific and relatively predictive GSMs can be derived automatically from genome sequences [8]. However, the main simulation methods for GSMs such as flux balance analysis (FBA) [9] and stochastic sampling [10] were originally developed for single species, not communities.

In single-species FBA, a key assumption is that the simulated species optimizes its fitness (e.g., growth). This can be interpreted as a decision making problem where the organism needs to optimally control its (evolved) metabolic network. However, in co-culture, the degree to which one species reaches its objective may depend on the metabolic activity of all species, for example, when species compete for nutrients. Dynamic FBA (dFBA) explicitly accounts for nutrient concentrations and thereby for such interactions; it combines the FBA principle with iterations over time to reflect changing environmental conditions [11]. Recently, also scalable methods for dFBA simulation of communities have been proposed [12]. Yet, a drawback of dFBA is that it requires reliable knowledge on the form and parameters of uptake kinetics, or at least on how bounds on uptake kinetics depend on environmental conditions. This knowledge is hard to obtain and without it, the simulation results can be unreliable [5].

Incomplete information on uptake kinetics raises a new frontier in decision making for the simulation of interacting microbes in co-culture: the presence of multiple decision making entities with potentially conflicting objectives. For example, in d-OptCom, an influential method for dFBA of a community of GSMs, and in its metabolically stationary state sibling OptCom [13], decision making is modeled as a bi-level optimization problem. On one level, the community strives towards a fitness goal (high community biomass production) and on the other level each microbial species optimizes its own fitness (growth rate). Abstractly, there are two types of decision makers, one making community decisions and one making decisions for individuals. Note that the existence of an apparent community decision maker is hypothetical—it could result from species co-evolution [14, 15].

Because community and individual decision makers may follow contradictory strategies, a principle for conflict resolution is needed. Some possibilities used for GSMs are: the community strategy takes precedence over individual decision makers [13], a community strategy must be Pareto optimal for the individual decision makers [16], and a community strategy must be a Nash equilibrium for the individual decision makers [17]. In other methods, the emergence of multiple decision makers has stimulated the use of game theory for the analysis of microbial interactions [18].

Here, however, we emphasize an aspect of community modeling that is largely ignored in the corresponding literature: going from one to several microbial species, the interpretation of the metabolically stationary state assumption in constraint-based modeling depends on the type of cultivation environment. The two main environments for cultivating microbes are (assumed) chemostat and batch processes. For FBA-based analysis, their different concepts of metabolically stationary state lead to different models for *decision making*. In particular, assumptions on environment and decision making have fundamental impact on whether organisms in a community of GSMs can coexist or not, and at which quantitative microbial community composition.

Because these dependencies have not yet been investigated systematically, we formulate four methods for simulating metabolically stationary states, corresponding to combinations of batch and chemostat cultivation, and two different modes of microbial decision making, distributed (rational agent) and centralized (rational community). In these formulations, we put a novel emphasis on what information (local/global) the decision makers have access to. The combination steady state batch/rational community resembles the community FBA (cFBA) formulation [19, 20]; the chemostat formulations applicable to GSMs are new. We demonstrate the qualitative differences between the approaches on two toy-examples, a prisoners dilemma (PD) model for decision making and a nutrient limitation model for coexistence. As expected, switching from rational agent to rational community, PD switches from defection to cooperation. For nutrient limitation, the four models yield qualitatively different results. Furthermore, using a novel numerical scheme to handle models of realistic size, we apply the chemostat models to investigate the impact of decision making on a syntrophic community of amino acid auxotrophic *E. coli* mutants [21]. Unexpectedly, distributed, rather than centralized, decision making opens up a larger array of possible cooperative solutions.

## Results

### Chemostat versus batch environment

**Fig. 1:**
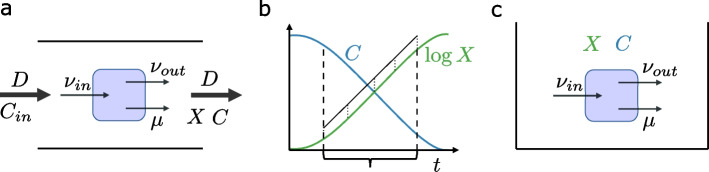
Cultivation systems and their implications for metabolically stationary state conditions. For definitions of mathematical variables, see Section Methods. **a** Chemostat as an open system in steady state. Black: time-constant entities; bold arrows: flows; normal arrows: metabolic fluxes; rounded rectangle: cell. **b** Dynamics in batch cultivation of cells with a phase of metabolically stationary state (constant specific growth rate, implying linear increase of the logarithm of the species concentration, insensitive to external concentrations) between dashed vertical lines. **c** Metabolically stationary state in the closed batch system (time-constant entities in black)

In a *chemostat* as an open system, a fluid flow (dilution rate *D*) adds nutrients (inflow concentrations $C_{in}$) and flushes out parts of the cultivation medium, keeping the cultivation volume constant (see Fig. 1a). A metabolically stationary state requires that metabolic fluxes ($\nu$), species abundances (*X*), and environmental nutrient concentrations (*C*) are constant over time (*t*). For the (non-zero) absolute microbial species abundances to be constant, the (specific) growth rates ($\mu$) must be equal to the dilution rate *D* (henceforth called D-growth).

Assuming growth maximization, the growth rate depends on the environmental nutrient concentrations via uptake kinetic functions that determine the upper bounds of uptake fluxes. In turn, environmental nutrient concentrations depend on fluxes and species abundances. When kinetic functions increase monotonically with environmental nutrient concentrations, negative feedback between biomass and nutrient concentrations inside the chemostat may give rise to nutrient-limited steady states [22]. Correspondingly, finding the steady-state biomass concentrations requires an explicit representation of extracellular substrate concentrations.

In a *batch* process as a closed system, all nutrients are provided at the beginning of the cultivation and nothing is flushed out (Fig. 1b,c). In contrast to a chemostat, with growing biomass, environmental nutrient concentrations are not constant over time, leading to different growth phases. A modeled metabolically stationary state therefore refers to the relaxed condition that metabolic fluxes as well as growth rates are time-constant. This typically holds during the exponential growth phase, which is used experimentally to determine growth rates. Throughout this manuscript, we call such a system a steady state batch (or, for short, batch if it is clear from the context).

A steady state batch process has two important implications: First, extracellular nutrient conditions are not limiting. Specifically, the relevant environmental nutrient concentrations are assumed to be in a regime where the kinetic functions determining the upper bounds of the growth limiting uptake fluxes are insensitive to the nutrient concentrations. Note that any system operating a metabolically stationary state under non-limiting extracellular nutrient conditions is equivalent to a steady state batch, without having to be a batch cultivation. Community models for such systems do not require a representation of environmental nutrient concentrations. Second, the relative species concentrations must be constant, implying that all species with non-zero abundance grow at the same rate averaged over time (henceforth called community steady state). This allows to properly model inter species crossfeeding of compounds and some GSM-based studies of communities apply community steady state [19, 20]. However, for non-interacting microbes in a batch, a community steady state will only occur if all concerned species have the exact same growth rate by chance, a situation that never happens in practice. Therefore, to simulate coexistence in a consortium, an explicit interaction between microbes, such as crossfeeding [20] or some form of *agreement* to grow at the same rate is mandatory. Other GSM-based methods do not impose a community steady state [13, 16, 17], thereby implicitly assuming a non-closed system. Overall, thus, the assumptions on the environment—implying observability of nutrient concentrations or lack of observability—also have implications for models of decision making in FBA-type analyses.

### Community models

To cover the two principal dimensions environment (chemostat vs batch) and decision making (rational agent vs rational community), we developed four models of microbial community growth at metabolically stationary state using metabolic networks. They are based on a general system of equations and differential equations governing the extracellular metabolite concentrations *C* and the absolute (relative) species concentrations *X* (*x*) for chemostat (for batch). Importantly, the common model includes the network stoichiometry as well as flux constraints. We assume intracellular (metabolically) stationary state as in FBA and dFBA [9, 23]. Then we impose assumptions about decision making that lead to the four specific models (see Methods for details).

For *rational agent models*, we assume that each cell is a decision making entity, using the extracellular concentrations as information to maximize its growth rate. As foundation for its decision making, each cell uses local information, in this case the extracellular compound concentrations, as well as its own flux constraints. This assumption seems intuitive for microbial species that do not share an evolutionary history of interactions. In the CA model (where ’C’ stands for chemostat and ’A’ for agent), the capacity constraints depend on the extracellular concentrations *C* through uptake kinetics. For the BA model (where ‘B’ stands for steady-state batch), contrary to practice in parts of the GSM consortium literature [5], the extracellular metabolite concentrations are constant and unknown to the agents. Under these conditions, community steady state is virtually impossible (see Methods). The BA model is therefore of little practical relevance and we included it only for completeness.

The *rational community models* assumes that, through a time of coexistence, a community has learned to optimize its (D- or balanced-) growth rate while cooperating to create a favourable nutrient environment. Note that what the community wants to achieve through cooperation, and with it the formal community objective function, may vary. A biologically relevant community objective, so far not formulated as FBA objective, is that in many parasitic consortia, the parasite wants to optimize growth without killing the host, which needs cooperation [24]. Here, for simplicity and in line with the literature [5], we consider only the community objective of maximizing total biomass production.

For the rational community chemostat (CC) model, we assume that the community has knowledge of and power over the global cellular exchanges of compounds (see Methods). Since different community decisions may benefit different organisms (in terms of species abundances and other factors), having a range of community optimal strategies in terms of fluxes and extracellular concentrations, but given different species abundances, it is not possible to know which strategy the community will settle for without detailed knowledge of the ‘negotiation’ process leading up to a decision. Thus, the step from rational agent to rational community is not about assuming full knowledge of how the community decides, but that actively influencing the extracellular metabolite concentrations is taken into account in its decision, while optimizing some assumed objective. In the steady state batch rational community (BC) case, since there is no explicit representation of the extracellular metabolite concentrations, the community decision maker can only take global constrains on uptakes by the community into account (see Methods).

Importantly, the optimization problems involved in all models lend themselves to a symbolic reformulation via Karush Kuhn Tucker (KKT) [25] conditions for solving the models. When this approach is feasible (depending on model size), it has the advantage of identifying all optima. For larger chemostat models, we devised a numerical optimization approach via a Mixed Integer Linear Program (MILP) formulation, for which efficient solvers exist (see Methods for details).

### Prisoners dilemma

As a first test for our community models, we used the metabolic network setting of the so-called called Prisoners Dilemma (PD) [26] game theory example from [17], which makes differences in conflict resolution mechanisms concrete. PD is a two player symmetric game with payoffs shown in Table 1. Mutual cooperation generates the largest overall benefit, but defection by one player yields a higher payoff for this player if the other player cooperates.

**Table 1:** Generic prisoners dilemma payoff matrix (numbers unrelated to Fig. 2). The first and second number in the round brackets denote the payoffs for player 1 and 2, respectively.

Player 2	Player 1	
	Cooperate	Defect
Cooperate	(3, 3)	(1, 4)
Defect	(4, 1)	(2, 2)

A biological PD may be yeast cells feeding off sucrose [17]. Sucrose is hydrolyzed to glucose and fructose extracellularly by the enzyme invertase. It is expected that producing and secreting invertase comes at a metabolic cost. However, it may also give a growth benefit, if being an invertase producer means that more sugars will be hydrolyzed close to the producer. If the cost is relatively high and the benefit relatively low, cheating by producing no invertase becomes a desirable strategy.

**Fig. 2:**
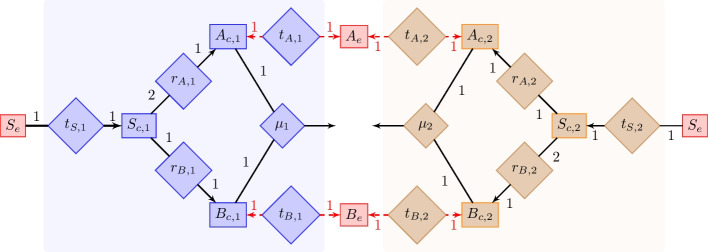
A PD microbial consortium [17]. The network is shown as a bipartite graph with metabolites (rectangles; red means extracellular) and reactions (diamonds). For turnover of a reaction, all its adjacent metabolites (indicated by edges) are used / produced jointly. Numbers next to edges are stoichiometric coefficients. The directionality of a reaction is determined by the arrows on the edges: arrows indicate products, and consequently arrows on both sides of a reaction denote bidirectionality. For example, $t_{A,1}$, the transport reaction of *A* in organism 1, is bidirectional. Likewise, the representation of $\mu _1$, the growth reaction of organism 1, specifies that the reaction is uni-directional and uses one unit of $A_{c,1}$ and one unit of $B_{c,1}$ to produce biomass. The subscripts *c* and *e* denote intra- and extracellular compounds, respectively. Species 1 and 2 (blue and brown symbols) can choose to crossfeed the compounds *A* and *B* to increase their yields by activating the reactions with the red dashed lines

An abstracted metabolic community version of PD is shown in Fig. 2. Species 1 and 2 both have the capacity to produce metabolites *A* and *B* and need both to grow, but species 1 produces *A* and species 2 produces *B* at lower yield than the other. Thus, for the community, mutual cooperation (crossfeeding) will lead to the highest biomass yield, whereas for the individual species, the highest yield is obtained by not secreting anything, while still being fed by the other species. The scenario, thus, requires conflict resolution: it pits the community and individual decision makers against each other.

To explore decision making in CA, CC and BC models of the metabolic PD system in Fig. 2, we were interested in whether, using a specific simulation model, the community achieves a fitness bonus by utilizing crossfeeding or whether the organisms refuse to cooperate. Quantitative simulation results are shown in Table 2. Apart from these symmetric (non-zero) solutions, non-symmetric solutions where one species has zero abundance occur. We do not consider these solutions without potential for cooperation here.

**Table 2:** Flux values of PD simulations for CA, CC and BC

Variable	CA	CC	BC	CC $D=1.2$
$C_{A_e}$	0	0.5		0
$C_{B_e}$	0	0.5		0
$C_{S_e}$	1.5	1.13		3.6
$X_1$	1.42	1.97	0.5	1.07
$X_2$	1.42	1.97	0.5	1.07
$\nu _{t_S, 1}$	1.5	1.13	10	3.6
$\nu _{t_A, 1}$	0	0.5	5	0
$\nu _{t_B, 1}$	0	− 0.627	-5	0
$\nu _{r_A, 1}$	0.5	0	0	1.2
$\nu _{r_B,1}$	0.5	1.13	10	1.2
$\nu _{\mu , 1}$	0.5	0.5	5	1.2
$\nu _{t_S, 2}$	1.5	1.13	10	3.6
$\nu _{t_A, 2}$	0	− 0.627	− 5	0
$\nu _{t_B, 2}$	0	0.5	5	0
$\nu _{r_A, 2}$	0.5	1.13	10	1.2
$\nu _{r_B, 2}$	0.5	0	0	1.2
$\nu _{\mu , 2}$	0.5	0.5	5	1.2

Variable names correspond to the named reactions and compounds in Fig. 2, identified by subscripts to $\nu$ for fluxes and to *C* for concentrations. For CA and CC simulations, we set the inflow nutrient concentration mixture to $C_{in, A_e}=0$, $C_{in, B_e}=0$ and $C_{in, S_e}=10$. As capacity constraints, the uptake fluxes of both organisms, defined in the uptake direction, were assumed to be smaller than their respective extracelleluar concentrations, $\nu _{t_S} \le C_{S_e}, \nu _{t_A} \le C_{A_e}, \nu _{t_B} \le C_{B_e}$ (organism subscripts on the fluxes omitted). For BC, we used the culture uptake bounds $u_{A_e}=0$, $u_{B_e}=0$ and $u_{S_e}=10$. The flow rate was set to $D=0.5$ except for the last column that used a higher flow rate, $D=1.2$

As expected, without a joint objective for the organisms, CA finds no crossfeeding. CC and BC find crossfeeding solutions, but these solutions differ. In CC, the secretion fluxes are greater than the uptake fluxes because some of the secreted material will be flushed out of the chemostat, rather than taken up by another organism. This generally makes crossfeeding in chemostats less attractive. For example, when increasing the flow rate *D*, the benefit of crossfeeding vanishes and CC switches to a solution without crossfeeding. In BC, void of an active out flush mechanism, all secreted material is taken up. Prior analyses of this PD focused only on conflict resolution mechanisms: the Nash equilibrium mechanism suggested in [17] results in no crossfeeding, whereas giving the community decision maker precedence [13] yields crossfeeding. Our results demonstrate how, in addition, assumptions on the environment influence crossfeeding predictions qualitatively as well as quantitatively.

### Coexistence microbial consortium

**Fig. 3:**
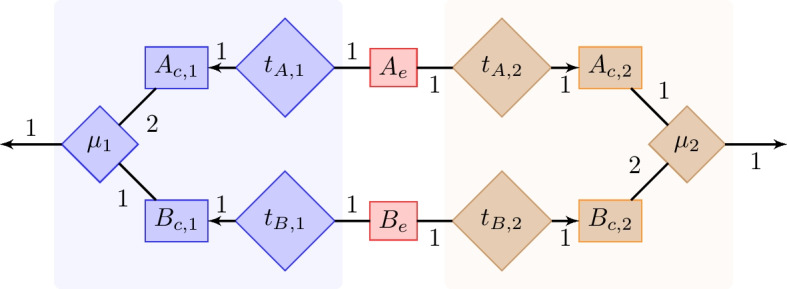
A coexistence microbial consortium of species 1 (blue) and species 2 (brown). Rectangles are metabolites and diamonds are reactions. Red rectangles are extracellular metabolites. The subscripts *c* and *e* denotes intra- and extracellular compounds, respectively. Numbers next to lines are stoichiometric coefficients

In a chemostat with two supplied nutrients, coexistence of two distinct species may emerge if, depending on the supply concentrations, the species reach a state in which they are limited by different nutrients [27, 28]. This (potentially competitive) coexistence does not rely on direct interactions such as crossfeeding. For the CA, CC and BC models, we investigated under what circumstances coexistence emerges for the non-crossfeeding metabolic network models in Fig. 3. There, both species need both externally supplied compounds $A_e$ and $B_e$ to grow, but because of their different network stoichiometries, species 1 needs more of compound $A_c$ and species 2 needs more of compound $B_c$.

For CA and CC, we varied the nutrient composition of the inflow, ($C_{in, A_e}, C_{in, B_e}$), linearly from (0, 10) to (10, 0). We set the flow rate $D=1$ and the uptake flux limitations to twice the corresponding substrate concentrations ($\nu _{t_{A},i} \le 2\cdot C_{A_e}$ and $\nu _{t_{B},i} \le 2\cdot C_{B_e}$; symbols defined in Fig. 3).

**Fig. 4:**
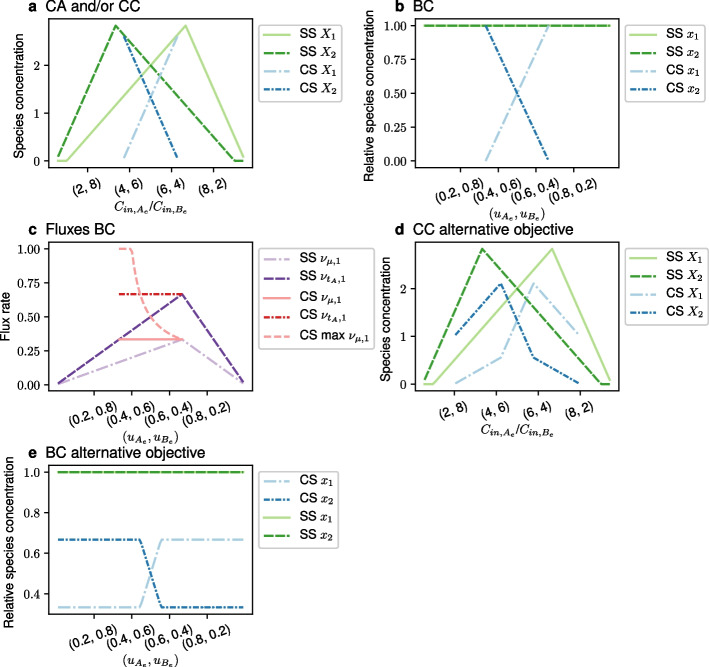
Coexistence results for the network in Fig. 3 for varying environmental conditions using CA, CC and BC. Abbreviations: SS - single-strain solution shows the value of a variable for one species, while the other species has zero biomass. CS - coexistence solution shows the value of a variable for one species, while for the other species, the value of the same variable is given by the other CS curve. **a** Species concentrations *X* for changing supply mixtures $C_{in}$ in CA or CC; they yield identical solutions. **b** Relative species concentrations *x* for changing input flux mixtures *u* for BC. SS curves for the two species coincide. **c** Selected fluxes of species 1 for changing input flux mixtures *u* for BC. **d** Same information as in (a), but only using CC and with an alternative objective function. **e** Same information as in (b), but with an alternative objective function

Lacking a potential to crossfeed, CA and CC generated identical results. Figure 4a shows identical, horizontally mirrored, single-strain solutions, that is, solutions where only one species exists. The single-strain solution of species 1 starts flat at zero, which is a regime where the concentration of $C_{A_e}$ is so low that species 1 cannot grow at the flow rate $(D=1)$; it is flushed out of the chemostat. After the zero regime comes a regime in which the growth rate of species 1 is limited by $C_{A_e}$ and the concentration of species 1 increases linearly with $C_{in, A_e}$. This continues with increasing $C_{in, A_e}$ and decreasing $C_{in, B_e}$, until $C_{B_e}$ becomes growth limiting, and the species concentration goes down linearly. A coexistence solution (CS) exists in one central regime, throughout which species 1 is limited by $C_{A_e}$ and species 2 is limited by $C_{B_e}$. At the concentration mixture where the dark blue curve (CS) goes to zero and ends, the light blue curve (CS) touches the light green curve (SS). At this point, where the lower coexistence solution goes to zero, the upper coexistence solution becomes a single-strain solution.

For BC, we varied the culture uptake bounds $$u_{A_e}$$ and $$u_{B_e}$$, which constrain the total uptake flux of the nutrients *A* and *B* of all consortium members together (see Methods for details). Specifically, we varied ($$u_{A_e}, u_{B_e})$$ linearly from (0, 1) to (1, 0). The individual community member’ uptake bounds were $$\nu _{t_{A},i} \le 2$$ and $$\nu _{t_{B},i} \le 2$$, where index *i* denotes the species. The main distinction from the chemostat scenario is that in BC, the single-strain solutions are identically one (Fig. 4b), due to the relative species concentrations.

Despite the apparent similarity between CA and BC, the interpretation of the coexistence solutions differs. For CA, a coexistence solution emerges without interspecies *communication*, simply because, at the species level, the growth rates of species 1 and 2 are limited by the uptake rates of $A_e$ and $B_e$, respectively. This is a known result from chemostat modelling [22]. Thus, at their steady state concentrations, the species reach a self stabilizing equilibrium, where neither species can grow faster than the assumed $D=1$.

In contrast, the growth rates of the species in the coexistence solution of BC (Fig. 4b) are not restricted by individual species uptake fluxes. Figure 4c shows for species 1 (a horizontal mirror image of species 2) that the uptake flux $\nu _{t_{A}, 1}$ always remains below its upper bound of 2. Instead, the growth rates are restricted by the global culture uptake bounds *u*. With regard to these constraints, the community steady state solutions, where the species grow at the same rate, are not the only solutions. As shown in Fig. 4c, in the coexistence solution, species 1 voluntarily grows at a rate that is lower than its maximal growth rate (CS max $\nu _{\mu , 1}$ exceeds the take-all single-strain solution $\nu _{\mu , 1}$ since it is operating at a lower relative species concentration). If the species did not *communicate* that growing at the same rate maximizes community biomass production, single species would claim more resources for themselves and break the metabolically stationary state. Thus, the coexistence solution we see is a result of the objective function.

To elucidate the dependence of the coexistence solutions of CC and BC on the community objective function, we changed the objective of CC and BC to maximizing the sum of growth rates, $\sum _{i} \nu _{\mu , i}\delta (X_i>0)$, rather than total community biomass production, $\sum _{i} \nu _{\mu , i}X_i$ ($X_i$ is replaced by $x_i$ in BC). Figure 4d-e shows that the changed objective function results in coexistence solutions that differ from the ones in Fig. 4a-b. This reflects the well-known impact of objective function choices on GSM-based predictions, and in addition underlines our arguments about the importance of assumptions on the environment.

### Synthetic community of amino acid auxotrophic *E. coli* mutants

In an early example of engineered microbial communities, Wintermute and Silver [21] showed that *E. coli* mutants that are auxotrophic for single (different) amino acids would sometimes show co-growth in pairs in amino acid-free medium. This implies that the strains somehow supply each other with the required amino acid. This phenomenon termed syntrophy prompted follow-up contributions, both experimental [29] and theoretical [17, 30]. According to our quantitative criteria, 10 out of 91 of the strain pairs investigated by Mee et. al. [29] grew syntrophically (see Methods for details). Given that these synthetic communities require microbial interaction for growth, we chose them to test our approach on a biologically realistic example, with more realistic, larger-scale models. Specifically, we aimed to compare the decision making models in the chemostat setting.

We implemented an amino acid syntrophic community in the core model for *E. coli* model from the BIGG repository of metabolic network models [31] (model e_coli_core with 72 metabolites and 95 reactions). This core model includes only the amino acids glutamine and glutamate. Since glutamine is derived from glutamate, glutamate/glutamine deletion mutants would not make up a feasible syntrophic community. We therefore created an alanine/glutamine syntrophic community by adding alanine and its synthesis from glutamate to the core model. We denote the alanine and glutamine auxotrophic strains as ala-aux and gln-aux, respectively. To obtain solutions with and without co-growth, small amounts of alanine and glutamine were added to the simulated medium, setting a dilution rate $D=0.1$ (see Methods for details). Details about the specification of the syntrophic community model and the used simulation parameters are given in Additional file 1.

We can characterize and analyze the CC and CA models in the two-dimensional biomass concentration space of steady-state solutions. The governing principle is that, at any point in this space, the strains are growing as fast as they can—either individually or as a community for CA and CC, respectively. If that means growing faster or slower than $D=0.1$, no feasible solution ensues. Thus, solutions are only found for non-growing strains (zero biomass) or strains that have a biomass concentration for which the supply of some extracellular metabolite limits the growth rate to 0.1. For our models, the strains may either be limited by the supply of the amino acid for which they are auxotrophic, or by the glucose supply.

**Fig. 5:**
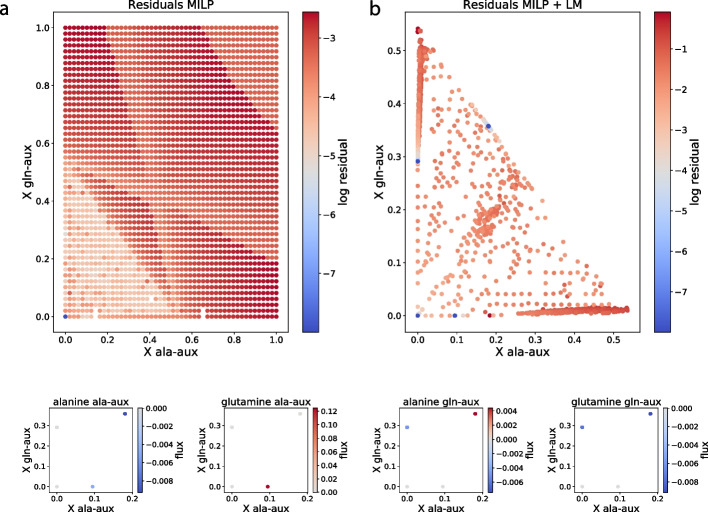
Syntrophic community in a chemostat with rational community model. (**a**)–(**b**): Maximal constraint violation and position in *X* space for 2500 starting points from a linearly spaced grid $[0, 1] \times [0, 1]$. **a** shows the minimal residuals found by the MILP solver. **b** shows (final) constraint violations after using each point in (a) as a starting point for minimizing the residual with the Levenberg-Marquardt algorithm (MILP + LM). Violations smaller than $10^{-8}$ are truncated to $10^{-8}$. **c**: Exchange of extracellular metabolites on a per strain, per solution basis. The values are net metabolite exchanges, scaled with the biomass concentration of the respective strain. Negative values means uptake. Only solutions with violations below $10^{-8}$ are shown

For CC, Fig. 5a shows the minimal residual absolute constraint violations when fixing the biomass concentrations to a regular grid. Sometimes, the MILP solver hit its imposed time limit, introducing irregularities to the otherwise smooth color field. Relaxing the constraint on the biomass concentrations, each solution in Fig. 5a was used as a starting point for an iterative optimizer that minimized constraint violations, giving the solutions in Fig. 5b (see Methods for details).

This results in four distinct solutions in biomass concentration space that have significantly lower residual than the other solutions and can be expected to be feasible. Three solution involve no actual communities: The trivial solution, for which no strain grows and two single-strain solutions where one strain is zero and the other strain grows, limited by the supply of the amino acid it depends on. Lastly, the upper right coexistence solution is no longer limited by amino acids, but instead by glucose. The metabolic exchange fluxes in Fig. 5c show that, by crossfeeding, the cooperating strains generate as much amino acids as they need, as long as they have glucose.

**Fig. 6:**
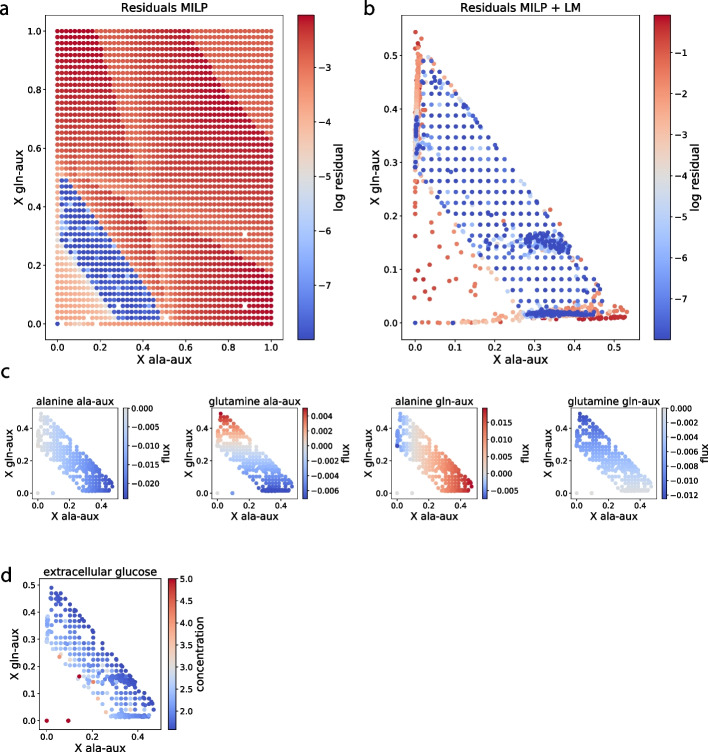
Syntrophic community in a chemostat with rational agent model. (**a**)–(**b**): Maximal constraint violation and position in *X* space for 2500 starting points, as in Fig. 5ab. **c**: Exchange of extracellular metabolites on a per strain, per solution basis as in Fig. 5c. Negative values mean uptake. **d**: Extracellular glucose concentration on a per solution basis. The color scale is truncated upwards to make the gradient visible

The corresponding results for CA (Fig. 6b) include the trivial solution (no biomass), the two single-strain solutions and the coexistence solution of CC. In addition, we find a band of solutions in which all points in *X* space have a feasible coexistence solution. Despite that CA has no explicit mechanism for cooperation, many of the solutions in the band feature amino acid crossfeeding. As a consequence of this, ala-aux achieves a higher biomass concentration than in its single-strain solution.

The apparent crossfeeding emerges because of glucose excess; the strains may allocate glucose wastefully without compromising their own growth rate (objective in CA) since amino acid availability limits the growth rate. One such wasteful glucose allocation strategy is to secrete amino acids, leading to crossfeeding. The location of a cooperative solution in the solutions band depends on two phenomena: First, crossfeeding, in which a strain supplies the other strain with the amino acid it cannot produce, but also, second, competition, in which a strain partly depletes an amino acid for the other strain. Figure 6c shows that, in all coexistence solution where the biomass concentration of gln-aux is higher than in its single-strain solution, ala-aux secretes glutamine (crossfeeding). Analogously, when gln-aux biomass is lower than in its single-strain solution, ala-aux consumes gln (competition). Switching the roles of the strains, the same holds when ala-aux is higher and lower than its single-strain solution biomass concentration.

Finally, the solution band in Fig. 6b is limited by an upper and a lower diagonal. The upper diagonal corresponds to a glucose limitation. As seen in Fig. 6d, at the diagonal, the extracellular glucose concentration approaches the value 1.58, the lowest concentration at which the cells can still maintain the growth rate 0.1 (approximately, see Additional file 1). The lower diagonal corresponds to a competition limit; below it, even if the strains compete maximally for amino acids, at least one of them will still grow faster than $D=0.1$. Hence, multiple solutions for coexistence can be explained by the interplay of different mechanisms, together with decision making afforded by the internal degrees of freedom afforded by the metabolic network.

A technical observation is that the solutions band in Fig. 6b contains two kinds of solutions: those located on a regular grid and irregularly placed ones. The regularly located solutions correspond to Fig. 6a; they were found as feasible already by the MILP solver and directly transferred. The red points of Fig. 6a were handed to the Levenberg-Marquard solver, aiming at lowering their residuals, while relaxing the requirement of staying on the grid (see Section Methods). In this step, the solutions migrate across biomass concentration space. Some solutions reach acceptable residuals ($<10^{-8}$) inside the solutions band, but typically not on the grid points.

## Discussion

Our study draws heavily on the long tradition of chemostat community models that take the extracellular environment into account [22, 27]. Key results of chemostat analysis are that, with constant nutrient concentrations in the feed, competing species may coexist under D-growth, if they are limited by different nutrients [28]. This enables models with (potentially multiple valid) coexistence states originating from both crossfeeding and differential nutrient limitations [32]. However, these models do not incorporate intracellular metabolic networks with degrees of freedom in establishing fluxes, and thus no decision making. dFBA and related approaches incorporate both. However, the benefit of compute-intensive calculation of transient metabolic states by dFBA is questionable: To be accurate, it requires explicit uptake kinetics to represent species interactions via the extracellular environment. If only metabolically steady states are requested, which is often the case [14, 17], how to deal with multiple steady states using dFBA is unclear. The present work aims to combine the two worlds of chemostats and FBA/GSMs, to capture all potential steady states without computing transient states.

To incorporate FBA models in the chemostat community model framework, we accounted for the fundamentally game theoretic problem of multiple decision makers [18]. In line with previous proposals for community modeling, we explored two flavors: rational agent and rational community. For our rational community models, we allowed the community to optimize both its shared metabolism and the environmental nutrient concentrations to achieve a community objective. However, we did not explicitly optimize the species concentration variables. This acknowledges that, if different species concentrations favor different species, and thereby yield multiple optima in terms of fluxes and nutrient concentrations, we do not know which optimum the community would choose.

One would expect that a community’s decision depends on the overarching framework and on the particular objective imposed. For example, by maximizing biomass production of the community, crossfeeding emerges in the PD scenario. However, our community models demonstrate that also environmental variables play a role. For example, by increasing the flow rate in the chemostat, the benefit of crossfeeding decreased, so that rational communities abolished crossfeeding. This phenomenon might be relevant for the gut microbiome, where the significance of other aspects of flow has been investigated [33].

Our models also suggest that coexistence in batch (BC) relies on a different mechanism than in chemostat (CA and CC). In BC, the community steady state is not a consequence of nutrient limitations caused by community growth. Coexistence requires *agreement* to coexist in the community, without any external enforcement mechanism, contrary to the chemostat models. Agreement without enforcement may amount to *forced altruism*, a modelling artifact discussed in detail in the context of PD by Chan et. al. [20]. The emergence of forced altruism in terms of coexistence at community steady state, rather than in terms of crossfeeding, is to our knowledge a new perspective that may be relevant for future community simulation methods.

For the amino acid auxotrophic mutants, a surprising result was that the non-cooperating chemostat model CA found many more cooperative solutions than the chemostat model CC that enforces cooperation. Specifically, we found a band of cooperative and competitive solutions in biomass concentration space. The emergence of this band depended on the strains being amino acid, rather than glucose (energy) limited, leaving the models many degrees of freedom even when maximizing their growth rate.

Experimentally, 10 (out of 91) pairs of single amino acid deletion mutants showed syntrophic growth [29]. Our results suggest that the mutants do not crossfeed amino acids in a targeted way based on the partners’ needs, but instead both secrete an array of amino acids. Co-growth would then occur spontaneously when the arrays of secreted amino acids happen to include the auxotrophies of both mutants. A hypothetical mechanism for secretion of amino acids in this case is that, when a mutant strain reaches a synthetic amino acid starvation, lacking an evolved regulatory response, some amino acids may start enriching. To validate such predictions experimentally, one needs metabolomics data for amino acid deletion mutants growing under starvation for the respective amino acid. Unfortunately, only one corresponding experiment is available in the literature, and this analysis of histidine auxotrophic *E. coli* measured only intracellular metabolomics [34]. The dataset is uninformative for our amino acid secretion hypothesis, and conclusions on the subject require targeted experimentation.

The most important methodological finding for the synthetic community is that CA yields multiple non-trivial solutions in biomass concentration space. The band of stationary solutions implies that, approaching the problem with a dFBA chemostat model directly, one of the stationary states found here will be reached. Which state dFBA finds will depend on the initial state, but also on which FBA solutions the dFBA algorithm chooses in each update step, acknowledging that the FBA problem may have several distinct solutions. Hence, a single dFBA simulation is in itself of little value and using dFBA to uncover the full solution band seems difficult compared to CA.

A topic we only touched upon is the effect of the specific objective function choices on model predictions, which is a central one for GSM analysis. We believe that the qualitative results of PD are relatively robust to changes in the community objective, such as switching to a sum of growth rates objective. Contrarily, for the coexistence example, we saw that changing the community objective function gave a new set of coexistence states.

Lastly, in the chemostat literature [27], stability of stationary solutions of ODEs is a central topic, which we did not address. If we assume that the microbial species can make decisions and actively uphold a state or an equilibrium, exactly what stability means in this scenario may need additional theoretical attention. Such concepts may be important to evaluate resistance of microbial communities to invasion by pathogens.

## Conclusions

The analysis of GSMs has become a standard approach for the *in-silico* exploration of microbial communities. The step from simulating a single species, to simulating a community, however, adds challenges in terms of microbial decision making, but also with regard to how the cultivation environment is modelled. We expect that our results on applications ranging from prototypical game theory scenarios to realistic communities illustrate the importance of considering decision making and environment jointly.

## Methods

### General consortium models

The organisms in all the considered environment and decision making scenarios are represented by steady state constraint based models. The vector of metabolic fluxes (reaction rates) of microbial species *i* is denoted $\nu _i \in \mathcal {R}^{n_{\nu _i}}$. One element of each flux vector $\nu _i$ is the (specific) growth rate $\nu _{\mu , i}$. Modelling reactions between $n_S$ intracellular compounds at constant concentrations, intracellular stationary state introduces a stoichiometric matrix ${S_i\in \mathcal {R}^{n_S \times n_{\nu _i}}}$ for which it holds that

1 $$\begin{aligned} S_i\nu _i=0,\ \forall i \;. \end{aligned}$$

Furthermore, for some matrix $A_i \in \mathcal {R}^{n_A \times n_{\nu _i}}$ and a vector $b_i \in \mathcal {R}^{n_A}$, the fluxes have capacity constraints

2 $$\begin{aligned} A_i\nu _i \le b_i,\ \forall i\ . \end{aligned}$$

In case the index *i* corresponds to an uptake reaction, $b_i$ may be a function of the extracellular concentrations of the metabolites that the reaction takes up.

For the chemostat, we are interested in the steady state of the extracellular compound concentrations $C \ge 0\in \mathcal {R}^{n_C}$ (we denote dimensionalities of variable *x* by $n_x$) and the organism concentrations $X \ge 0 \in \mathcal {R}^{n_X}$ (see also Fig. 1). We consider a system with constant and equal inflow and outflow rates (dilution rate) *D*. The inflow has nutrient concentrations $C_{in}\in \mathcal {R}^{n_C}$. The matrix $T_i \in \mathcal {R}^{n_C \times n_{\nu _i}}$ maps reactions to exchanges of extracellular compounds. Assuming that compounds and cells are flushed out at a rate proportional to their concentrations, the dynamics of *C* and *X* are described by:

3 $$\begin{aligned} \frac{dC}{dt}= & {} D(C_{in}-C) - \sum _{i} T_i \nu _i X_i \end{aligned}$$

4 $$\begin{aligned} \frac{dX_i}{dt}= & {} X_i(\nu _{\mu , i} - D),\ \forall i \;. \end{aligned}$$

For steady state, we consider the case where the left-hand sides of the system of ordinary differential equations (ODEs) Eqs. (3-4) are zero and Eqs. (1-2) hold. In contrast, in steady state batch, the extracellular compound concentrations are assumed to have no influence on the fluxes. To avoid infinite uptakes, flux exchanges with the environment, modeled by changes in *C* in Eq. (3), are captured by a vector of culture uptake bounds, $u \in \mathcal {R}^{n_C}$. The species concentrations *X* are exchanged for the relative species concentrations *x*. The change to relative species concentrations allows them to stay constant over time under community steady state. To represent community steady state, a community growth rate $\nu _{\mu }^{\star }$ is introduced. In combination, the steady state batch system is then:

5 $$\begin{aligned} {\begin{matrix} u - \sum _{i} T_i \nu _i \cdot x_i \ge 0\\ S_i\nu _i=0,\ \forall i \\ A_i\nu _i \le b_i,\ \forall i\\ x_i (\nu _{\mu }^{\star }-\nu _{\mu , i}) = 0, \forall i\\ \sum _{i} x_i = 1\ . \end{matrix}} \end{aligned}$$

We use these formulations of chemostat and batch in metabolically stationary state to introduce four models. For ease of comparison, all model equations, plus extra information such as Karush Kuhn Tucker (KKT) [25] conditions used for solving the models, can be seen side-by-side in Additional file 1: Table S3.

### Rational agents models

Based on the assumption that each organism adjusts its fluxes to optimize its growth rate, and denoting variables resulting from the optimization problem with hat notation $\hat{\nu _i}$, the chemostat (CA) model is:

6 $$\begin{aligned} {\begin{matrix} D(C_{in}-C) - \sum _{i} T_i \hat{\nu }_i(C) X_i = 0\\ X_i(D -\hat{\nu }_{\mu , i}(C)) = 0,\ \forall i\\ C, X \ge 0\\ \hat{\nu }_i(C) = \mathop {\textrm{argmax}}\limits _{\nu _i\in \mathcal {R}^{n_{\nu _i}}} \nu _{\mu , i}, \forall i\\ s.t.\ S_i \nu _i=0,\ \forall i\\ A_i \nu _i \le b_i(C),\ \forall i \;. \end{matrix}} \end{aligned}$$

Note that without implementing a concentration dependency of the capacity constraints via *b*(*C*), the optimization problem is independent of the substrate and organism concentrations. This means that the modeled cells do not adapt their growth to changes in extracellular nutrient concentrations (no negative feedback possible). In most cases, this will imply that no solution will fulfill the D-growth requirement and only the trivial solution $X=0$ will be feasible.

Correspondingly, the steady state batch rational agent (BA) system is:

7 $$\begin{aligned} {\begin{matrix} u - \sum _{i} T_i\hat{\nu }_i \cdot x_i \ge 0 \\ x_i (\nu _{\mu }^{\star }-\hat{\nu }_{\mu , i}) = 0, \forall i \\ \sum _{i} x_i = 1\\ x \ge 0\\ \hat{\nu }_i = \mathop {\textrm{argmax}}\limits _{\nu _i\in \mathcal {R}^{n_{\nu _i}}} \nu _{\mu , i}, \forall i\\ s.t.\ S_i \nu _i=0,\ \forall i\\ A_i \nu _i \le b_i,\ \forall i \;. \end{matrix}} \end{aligned}$$

In this formulation, the rational agent assumption does not include the global equations (first lines) in the optimization problem. Furthermore, since the extracellular substrate concentrations are assumed to be constant, the optimization problem is independent of the first two lines of Eq. (7). In this scenario, community steady state is only possible for organisms that independently developed the exact same (condition dependent) maximal growth rate, a situation that will never occur in practice.

### Rational community models

To represent a chemostat environment and a rational community, using a concatenated flux variable $\nu = [\nu _1,,,\nu _{n_X}] \in \mathcal {R}^{n_{\nu }}$, the CC model reads:

8 $$\begin{aligned} {\begin{matrix} X_i(D -\hat{\nu }_{\mu , i}(X)) = 0, \forall i\\ X \ge 0\\ \hat{\nu }(X) = \mathop {\textrm{argmax}}\limits _{\nu \in \mathcal {R}^{n_{\nu }}, C\in \mathcal {R}^{n_C}} \sum _{i} \nu _{\mu , i}X_i\\ s.t.\ D(C_{in}-C) - \sum _{i} T_i \nu _i X_i = 0\\ S_i \nu _i=0,\ \forall i\\ A_i \nu _i \le b_i(C),\ \forall i\\ C\ge 0 \;. \end{matrix}} \end{aligned}$$

Note that, contrary to the rational agent models, Eq. (3) is now inside the optimization problem, and therefore, so are also all instances of the global variables *C*. Another important detail of the CC model is that the abundances *X* do not enter the optimization problem as optimization variables.

The BC model, as for the BA model, has no explicit representation of *C*, but different from the BA model, the community takes the macroscopic equation $u - \sum _{i} T_i\nu _i \cdot x_i \ge 0$ into account in the decision making process, leading to the system:

9 $$\begin{aligned} {\begin{matrix} x_i (\nu _{\mu }^{\star }-\hat{\nu }_{\mu , i}(x)) = 0, \forall i\\ \sum _{i} x_i = 1\\ x \ge 0\\ \hat{\nu }(x) = \mathop {\textrm{argmax}}\limits _{\nu \in \mathcal {R}^{n_{\nu }}} \sum _{i} \nu _{\mu , i}x_i\\ s.t.\ u - \sum _{i} T_i\nu _i \cdot x_i \ge 0 \\ S_i \nu _i=0,\ \forall i\\ A_i \nu _i \le b_i,\ \forall i \;. \end{matrix}} \end{aligned}$$

### Symbolic solutions

The models defined by Eqs. (6)–(9) are algebraic systems (if $b_i(C)$ is algebraic) with inner optimization problems. All computations in this manuscript were performed using a reformulation of the original systems using KKT, which turns Eqs. (6)-(9) into purely algebraic systems (Additional file 1: Table S3). Notice that the optimization problems in Eqs. (6)–(9) are all linear in the optimization arguments, implying that KKT provides sufficient conditions for global optimality [25]. The small examples (Fig. 2 and 3) were solved symbolically in Mathematica 9. Symbolic solutions have the advantage that we are confident that all solutions are found.

### Numerical approach to the chemostat models

For larger systems, prompted by computational infeasibility of the symbolic approach, we developed a numerical approach. As numerical methods applicable to Eq. 9 have been developed elsewhere [19, 20], we focus on the chemostat models.

Introducing the Lagrange multipliers $\lambda _1 \in \mathcal {R}^{n_A}$ for CA ($\lambda _1 \in \mathcal {R}^{n_A+n_C}$ for CC) and $\lambda _2 \in \mathcal {R}^{n_S}$ for CA ($\lambda _2 \in \mathcal {R}^{n_S+n_C}$ for CC), corresponding to inequality and equality constraints and an element wise product $\odot$, the algebraic, optimization free formulations of CA and CC ensue (Eqs. (10) and (11)) [25]. The last rows of (10) and (11) are the so-called complementary slackness conditions, assuring that, either an inequality constraint has to be an equality, or its corresponding Lagrange multiplier has to be zero. The multiplicative representation used in (10) and (11) is only one of many possible formulations of complementary slackness.

**CA**

10 $$\begin{gathered} D(C_{{in}} - C) \hfill \\ - \sum\limits_{i} {T_{i} } \nu _{i} X_{i} = 0 \hfill \\ X_{i} (D - \nu _{{i,\mu }} ) = 0,\forall i \hfill \\ C,X \ge 0 \hfill \\ S_{i} \nu _{i} = 0,\;\forall i \hfill \\ A_{i} \nu _{i} \le b_{i} (C),\;\forall i \hfill \\ \left[ {\begin{array}{*{20}c} 0 \\ \vdots \\ { - 1} \\ \end{array} } \right]^{T} + \lambda _{{i,1}}^{T} A_{i} + \lambda _{{i,2}}^{T} S_{i} = 0,\forall i \hfill \\ \lambda _{{i,1}} \ge 0,\forall i \hfill \\ \lambda _{{i,1}} \odot (A_{i} \nu _{i} - b_{i} (C)) = 0,\forall i \hfill \\ \end{gathered}$$

**CC**

11 $$\begin{gathered} D(C_{{in}} - C) - \sum\limits_{i} {T_{i} } \nu _{i} X_{i} = 0 \hfill \\ X_{i} (D - \nu _{{i,\mu }}) = 0,\forall i \hfill \\ C, X \ge 0 \hfill \\ S_{i} \nu _{i} = 0,\;\forall i \hfill \\ A_{i} \nu _{i} \le b_{i} (C),\;\forall i \hfill \\ \left[ {\begin{array}{*{20}c} 0 \\ \vdots \\ { - X_{1} } \\ 0 \\ \vdots \\ { - X_{{n_{X} }} } \\ 0 \\ \vdots \\ \end{array} } \right]^{T} + \lambda _{1}^{T} \left[ {\begin{array}{*{20}c} A & { - \frac{{db(C)}}{{dC}}} \\ 0 & { - I} \\ \end{array} } \right] \hfill \\ + \lambda _{2}^{T} \left[ {\begin{array}{*{20}c} S & 0 \\ { - \sum\limits_{i} {T_{i} } X_{i} } & { - ID} \\ \end{array} } \right] = 0,\forall i \hfill \\ \lambda _{1} \ge 0 \hfill \\ \lambda _{1} \odot \left[ {\begin{array}{*{20}c} {(A\nu - b(C))} \\ { - C} \\ \end{array} } \right] = 0 \hfill \\ \end{gathered}$$

Limiting $b_i(C)$ to be piece-wise linear concave functions, Eqs. (10) and (11) are quadratic systems, with potential to have many solutions. Querying the solutions that optimize some quadratic polynomial of the system variables ($\nu , X, C, \lambda$) results in a non-convex quadratic program, which can be solved directly with branching techniques; this includes guarantees of feasibility and global optimality [35]. The drawback with this approach is that it has no favorable bounds on the required computation time (NP-hard).

The non-convex quadratic program can be reduced to a more standard mixed integer linear problem (MILP), which still is NP-hard, but which has proven solvable for many problem instances with large numbers of variables [36]. To arrive at a MILP, for both (10) and (11), we (1) fix *X* to a grid and (2) make a linear formulation of the complementary slackness conditions. With this, no quadratic expressions remain.

Specifying a grid for the *X* vector and solving the system (10) or (11) for each grid point turns all quadratic expressions, except the complementary slackness conditions, into linear expressions. By fixing *X*, the equality constraints including *X* may no longer be feasible. For these equality constraints, two non-negative slack variables are introduced, one with positive and one with negative sign. With the slack variables, the system is feasible. Direct minimization of the slack variables gives the solution with minimal constraint violations.
(2) By introducing a vector of binary variables $\Delta$ with the same number of elements as $\lambda _1$, a large constant $\Omega$ and a variable *q* which is either equal to $A\nu - b(C)$ or $-C$, depending on which equation we consider, the linear complementary slackness conditions are:
  12 $$\begin{aligned} {\begin{matrix} 0 \le \lambda _1 \le \Omega \Delta \\ -\Omega (1 - \Delta ) \le q \le 0 \end{matrix}} \end{aligned}$$
  Note that $0 \le \lambda _1$ and $q \le 0$ are not introduced in Eq. (10), but exist already in Eqs. (10) and (11).

By introducing a grid in the *X* variable, we have traded the difficulty of solving a non-convex quadratic problem for solving many MILPs. The precision of the results obtained this way will depend on the granularity of the grid and thus the number of MILPs. Keeping the grid point density fixed, the number of MILPs increases exponentially with the number of organisms. A work around to avoid an intractable number of MILPs is utilizing a relatively sparse grid and refining the obtained solutions using gradient based optimization. Setting the objective as the sum of squares of constraint violations of system (10) or (11) the solutions were refined using the Levenberg-Marquardt algorithm [25]. Note that, for the refinement step, the optimizer may get stuck in a local minimum and never reach a feasible solution. For a solution to be feasible (and globally optimal), the maximal constraint violation should be virtually zero ($<10^{-8}$ used here).

### Quantitative criteria for syntrophic growth

Mee et. al. [29] investigated 14 single amino acid deletion mutants, leading to 91 pair experiments. The data provided for the growth experiments is in terms of measured fold increase on a per strain level; it captures by how much the optical density of a strain increases when paired with another strain, compared to when alone in the same medium. To state syntrophic growth, we required both strains to display at least a 10-fold increase.

## Supplementary Information

**Additional file 1**. Simulation parameters for the synthetic community and summary of mathematical model formulations.

## Data Availability

The code used to generate the data and figures for the PD, coexistence and syntrophic community examples is available at gitlab.com/csb.ethz/environment_fba.

## Abbreviations

BA: Batch model with agent decision makers
BC: Batch model with a community decision maker
CA: Chemostat model with agent decision makers
CC: Chemostat model with a community decision maker
FBA: Flux balance analysis
cFBA: Community flux balance analysis
dFBA: Dynamic flux balance analysis
GSM: Genome scale model
PD: Prisoners dilemma
SS: Single solution
CS: Coexistence solution
MILP: Mixed integer linear program
LM: Levenberg-Marquardt algorithm
KKT: Karush-Kuhn-Tucker
AA: Amino acid
