# Supplementary material for: Assumptions on decision making and environment can yield multiple steady states in microbial community models
Source: BMC Bioinformatics. 2023 Jun 22;24(Suppl 1):262. doi: 10.1186/s12859-023-05325-w (PMC10288676; doi:10.1186/s12859-023-05325-w)
Supplement: Supplementary file 1 — Additional file 1. Simulation parameters for the synthetic community and summary of mathematical model formulations. [file 12859_2023_5325_MOESM1_ESM.pdf]

Supplementary Material: Assumptions on decision making and environment can yield multiple steady states in microbial community models

Axel Theorell
axel.theorell@bsse.ethz.ch

Jörg Stelling
joerg.stelling@bsse.ethz.ch

April 26, 2022

Simulation Setup of the Synthetic Community of Amino Acid Auxotrophic *E. coli* mutants

Model creation

The community model was built around the stoichiometric model *e_coli_core* from the BIGG database [1]. For both the alanine and glutamine auxotrophic mutants (ala-aux, gln-aux), *e_coli_core* was extended with the reactions in Tab. S1.

Table S1: Reactions added to *e_coli_core*. BIGG [1] metabolite identifiers are used. Trailing e or c in the metabolite identifiers encode extra- and intracellular metabolites, respectively.

Reaction	Educts	Products	reversible
L-alanine transaminase	akg_c, ala__L_c	pyr_c, glu__L_c	Yes
L-alanine ABC transport	atp_c, ala__L_e, h20_c	adp_c, ala__L_e, h_c, pi_c	No
L-alanine secretion	ala__L_c	ala__L_e	No
L-glutamine secretion	gln__L_c	gln__L_e	No

Furthermore, for both ala-aux and gln-aux, to enforce an alanine dependency, alanine was added as educt to the biomass equation, requiring 0.513689 units of L-alanine per unit of biomass produced. The specific value comes from the biomass equation *BIOMASS_Ec_iML1515_core_75p37M* of the model *iML1515* [1].

For ala-aux and gln-aux, to mimic gene deletions, L-alanine transaminase (ALATA_L) and L-glutamine synthetase (GLNS) were removed, respectively.

Cultivation environment

The dilution rate D was set to 0.1. The metabolite concentrations in the feed used the values in Tab. S2. Oxygen, protons, ammonium, phosphate and water were seen as necessary, but not limiting, for respiratory growth and were given high concentrations (100). Glucose was seen as the major energy limitation and given an intermediate concentration (10). Low concentrations (0.1) were given to alanine and glutamine, to allow for for residual growth in the absence of the partner strain.

Uptake kinetics

All reversible transport reactions were separated into two irreversible transport reactions. Viewing the simulations as an exploration of mechanisms, rather than depicting a specific experimental scenario, the upper bounds on all uptake reactions were set to depend linearly on the concentration of all imported metabolites with linear coefficient 1. In case a transporter imports several compounds simultaneously, the compound with lowest concentration will be limiting.

Table S2: Metabolite concentrations in chemostat feed. BIGG [1] metabolite identifiers are used.

Metabolite	Concentration in feed
o2	100
h	100
nh4	100
pi	100
h2o	100
glc__D	10
gln__L	0.1
ala__L	0.1

Computation of theoretical glucose limitations

Using the chemostat rational agent model, *E. coli* deletion mutants showed a band of coexistence solutions (Fig. 5). Towards the upper diagonal of the band, Fig. 5d shows that the extracellular glucose concentration reaches a minimum value around 1.5. Due to the linear uptake kinetics with coefficient 1, the extracellular glucose concentration is also the upper bound of glucose uptake for both models. We investigate what the minimum glucose uptake is to decide whether the upper diagonal of the solutions band corresponds to a glucose limitation.

The ala-aux and gln-aux stoichiometric models described in section **Model creation** were merged into one stoichiometric community model, retaining separate intracellular and transport reactions, but sharing the extracellular compartment and exchange reactions. All uptakes except for the metabolites (using BIGG metabolite names) o2, h, pi, glc__D, nh4, h2o were set to zero, and the growth rates of both models were set to the dilution rate 0.1. The minimal glucose uptake of the community was 1.58, confirming that the upper diagonal indeed corresponds to glucose limitation. Note that the theoretical value presented here deviates slightly from the glucose limitation in the chemostat rational agent simulation since in this simulation, no extracellular alanine and glutamine were provided. Given their low concentrations, the deviation should be small.

Mathematical formulation of the models

Table S3: Models, Lagrangians and KKT formulations. For BA and CA, Lagrangian multipliers (inequality, λ_1, and equality, λ_2, multipliers) are introduced on a per species level (subscript i), whereas for BC and CC, global multipliers are introduced. Dimensionalities of multipliers vary between formulations. The symbol $\odot$ denotes element wise product. Rows #var and #EQ confirm that the numbers of unknowns and equations are equal.

	**BA**	**CA**	**BC**	**CC**
Eqs	$u - \sum_i T_i \hat{\nu}_i \cdot x_i \geq 0$<br>$x_i(\nu^\star_\mu - \hat{\nu}_{i,\mu}) = 0, \forall i$<br>$\sum_i x_i = 1$<br>$x \geq 0$<br>$\hat{\nu}_i = \underset{\nu_i \in \mathcal{R}^{n_{\nu_i}}}{\text{argmax}}\, \nu_{\mu,i}, \forall i$<br>$s.t.\ S_i \nu_i = 0,\ \forall i$<br>$A_i \nu_i \leq b_i,\ \forall i$	$D(C_{in} - C)$<br>$- \sum_i T_i \hat{\nu}_i(C) X_i = 0$<br>$X_i(D - \hat{\nu}_{i,\mu}(C)) = 0, \forall i$<br>$C, X \geq 0$<br>$\hat{\nu}_i(C) = \underset{\nu_i \in \mathcal{R}^{n_{\nu_i}}}{\text{argmax}}\, \nu_{\mu,i}, \forall i$<br>$s.t.\ S_i \nu_i = 0,\ \forall i$<br>$A_i \nu_i \leq b_i(C),\ \forall i$	$x_i(\nu^\star_\mu - \hat{\nu}_{i,\mu}(x)) = 0, \forall i,$<br>$\sum_i x_i = 1,$<br>$x \geq 0,$<br>$\hat{\nu}(x) = \underset{\nu \in \mathcal{R}^{n_\nu}}{\text{argmax}} \sum_i \nu_{\mu,i} x_i,$<br>$s.t.\ u - \sum_i T_i \nu_i \cdot x_i \geq 0,$<br>$S_i \nu_i = 0,\ \forall i,$<br>$A_i \nu_i \leq b_i,\ \forall i$	$X_i(D - \hat{\nu}_{i,\mu}(X)) = 0, \forall i$<br>$X \geq 0$<br>$\hat{\nu}(X) =$<br>$\underset{\nu \in \mathcal{R}^{n_\nu}, C \in \mathcal{R}^{n_C}}{\text{argmax}} \sum_i \nu_{\mu,i} X_i$<br>$s.t.\ D(C_{in} - C)$<br>$- \sum_i T_i \nu_i X_i = 0$<br>$S_i \nu_i = 0,\ \forall i$<br>$A_i \nu_i \leq b_i(C),\ \forall i$<br>$C \geq 0$
Lgr	$L_i(\nu_i) =$<br>$- \nu_{\mu,i} + \lambda_{i,1}^T (A_i \nu_i - b_i)$<br>$+ \lambda_{i,2}^T (S_i \nu_i), \forall i$	$L_i(\nu_i(C)) =$<br>$- \nu_{\mu,i} + \lambda_{i,1}^T (A_i \nu_i - b_i(C))$<br>$+ \lambda_{i,2}^T (S_i \nu_i), \forall i$	$L(\nu) =$<br>$- \sum_i \nu_{\mu,i} x_i$<br>$+ \lambda_1^T \begin{bmatrix} A\nu - b \\ -u + \sum_i T_i \nu_i x_i \end{bmatrix}$<br>$+ \lambda_{i,2}^T S_i$	$L([\nu, C]) =$<br>$- \sum_i \nu_{\mu,i} X_i$<br>$+ \lambda_1^T \begin{bmatrix} (A\nu - b(C)) \\ -C \end{bmatrix}$<br>$+ \lambda_2^T \begin{bmatrix} S \\ D(C_{supply} - C) \\ - \sum_i T_i \nu_i X_i \end{bmatrix}$
KKT	$\begin{bmatrix} 0 \\ \vdots \\ -1 \end{bmatrix}^T + \lambda_{i,1}^T A_i$<br>$+ \lambda_{i,2}^T S_i = 0, \forall i,$<br>$\lambda_{i,1} \geq 0, \forall i,$<br>$\lambda_{i,1} \odot (A_i \nu_i - b_i) = 0, \forall i$	$\begin{bmatrix} 0 \\ \vdots \\ -1 \end{bmatrix}^T + \lambda_{i,1}^T A_i$<br>$+ \lambda_{i,2}^T S_i = 0, \forall i,$<br>$\lambda_{i,1} \geq 0, \forall i,$<br>$\lambda_{i,1} \odot (A_i \nu_i - b_i(C)) = 0, \forall i$	$\begin{bmatrix} 0 \\ \vdots \\ -x_1 \\ 0 \\ \vdots \\ -x_{n_x} \end{bmatrix}^T$<br>$+ \lambda_1^T \begin{bmatrix} A \\ \sum_i T_i x_i \end{bmatrix}$<br>$+ \lambda_2^T S = 0, \forall i,$<br>$\lambda_1 \geq 0,$<br>$\lambda_1 \odot \begin{bmatrix} A\nu - b \\ -u + \sum_i T_i \nu_i x_i \end{bmatrix} = 0$	$\begin{bmatrix} 0 \\ \vdots \\ -X_1 \\ 0 \\ \vdots \\ -X_{n_X} \\ 0 \\ \vdots \end{bmatrix}^T$<br>$+ \lambda_1^T \begin{bmatrix} A & - \frac{db(C)}{dC} \\ 0 & - I \end{bmatrix}$<br>$+ \lambda_2^T \begin{bmatrix} S & 0 \\ - \sum_i T_i X_i & - ID \end{bmatrix}$<br>$= 0, \forall i,$<br>$\lambda_1 \geq 0,$<br>$\lambda_1 \odot \begin{bmatrix} (A\nu - b(C)) \\ -C \end{bmatrix} = 0$
#var	$1 + n_x + n_\nu + n_S + n_A$	$n_C + n_X + n_\nu + n_S + n_A$	$1 + n_x + n_C + n_\nu + n_S + n_A$	$3n_C + n_X + n_\nu + n_S + n_A$
#EQ	$n_x + 1 + n_S + n_\nu + n_A$	$n_C + n_X + n_S + n_\nu + n_A$	$n_x + 1 + n_S + n_\nu + n_C + n_A$	$n_X + n_C + n_S + n_\nu + n_C +$<br>$n_A + n_C$

References

[1] King, Z.A., Lu, J., Dräger, A., Miller, P., Federowicz, S., Lerman, J.A., Ebrahim, A., Palsson, B.O., Lewis, N.E.: Bigg models: A platform for integrating, standardizing and sharing genome-scale models. Nucleic acids research **44**(D1), 515–522 (2016)
